# Assessing variability among culturable phylloplane basidiomycetous yeasts from Italian agroecosystems

**DOI:** 10.1007/s11274-024-04147-z

**Published:** 2024-10-03

**Authors:** Matteo Ferluga, Michele Avesani, Marilinda Lorenzini, Giacomo Zapparoli

**Affiliations:** 1 Dipartimento di Biotecnologie, Università degli Studi di Verona, Strada Le Grazie 15, Verona, 37134 Italy; 2 Unione Italiana Vini, Viale del Lavoro 8, Verona, 37135 Italy

**Keywords:** Basidiomycetous yeasts, Agroecosystems, Fungicides, Extracellular enzymes, Microbial diversity

## Abstract

This study analysed basidiomycetous yeasts isolated from the phylloplane of crops and spontaneous plants in Italian agroecosystems. A total of 25 species belonging to 17 genera were recognized by analysing 83 isolates from vineyards and orchards, that are not treated with synthetic fungicides, and adjacent natural areas. *Rhodotorula graminis* and *Filobasidium magnum* were the most frequent species but 13 others were represented by a single isolate (e.g., *Buckleyzyma salicina*, *Pseudozyma prolifica*, and *Moniliella megachiliensis*). Preliminary analysis of (GTG)_5_-PCR fingerprinting revealed high genetic intraspecific heterogeneity. All isolates were characterized by their production of extracellular hydrolytic enzymes and their sensitivity to six commercial fungicides used in Italy. The isolates displayed great variability in these phenotypic traits, which play an important role in the survival of yeast populations in agroecosystems. Most of them exhibited lipolytic, proteolytic, *β*-glucosidase and pectinolytic activities, but only three (*F. magnum*, *Kwoniella mangroviensis* and *Ps. prolifica*) also had cellulolytic and amylolytic activity. Most isolates were sensitive to four fungicides, and one *R. graminis* isolate was resistant to all six. This heterogeneity was not related to the geographical origin of the isolates. The lack of selective factors (i.e. pesticide treatments) in the sampling fields and the presence of adjacent natural areas may have favored the maintenance of an elevated level of strain diversity. This study provides new information on phylloplane basidiomycetous yeasts in agroecosystems and opens the way to further investigations into the impact of agricultural practices on the microbial diversity of these natural habitats.

**Supplementary Information:**

The online version contains supplementary material available at 10.1007/s11274-024-04147-z.

## Introduction

Yeasts colonize the plant surfaces, such as leaves, flowers, fruits, branches and barks, that make up the phylloplane. In this environment, yeasts interact with other microorganisms, like bacteria and filamentous fungi, as they compete for nutrients (Sláviková et al. 2007; Kemler et al. 2017). Saprophytic yeasts, common on leaves and fruit, can compete with some fungal pathogens by affecting their growth, thereby exerting biocontrol effects (Freimoser et al. 2019). Moreover, the phylloplane harbors plant-growth promoting yeasts since they increase the nutrient availability for the host plant and induce a plant defense response against pathogens (Nimsi et al. 2023).

Since phylloplane contains limited nutrients, except in ripe fruits, and is exposed to solar radiation and temperature fluctuations, yeasts have developed genetic and physiological adaptations, including such as competing with host’s microbes, osmotic stress tolerance, pigmentations, the production of exopolysaccharides, hormone-like metabolites and exoenzymes in plants, which allow them to survive and grow in this environment. The ability to produce enzymes has been thoroughly investigated due to their potential biotechnological applications (Raveendran et al. 2018). Several hydrolytic enzymes (e.g., lipases, cellulases, pectinases and amylases) produced by yeasts isolated from the phylloplane are used in industrial and commercial processes (Da Silva et al. 2005; De Francesci et al. 2014; Thongekkaew et al. 2016; Haile and Ayele 2022; Sohail et al. 2022). The phylloplane therefore deserves investigation as a source for yeast-produced enzymes, which are required to improve and develop new biotechnological processes.

Phylloplane is colonized by taxonomically different groups of yeasts, the frequency of which depends on biotopes, geographical areas, plant species, the impact of human activity and other factors (Yurkov et al. 2015). Basidiomycetous yeasts are very common in leaves, fruit and the bark of plants in environments with zero or low human impact, such as forests, and industrial crops (Yurkov et al. 2015; Into et al. 2020). A considerable number of new species inhabiting the leaves of plants in pristine ecosystems (e.g., forest, tundra, desert) have been identified (Boekhout et al. 2022). These environments naturally excite much more interest than agroecosystems because they offer the possibility to survey undescribed microbiota. Surveys on microbial diversity in agricultural environments are therefore less attractive than unexplored natural habitats. Nevertheless, yeast ecology in agroecosystems is far from being completely understood. The practice of monoculture, which includes the use of fertilizers and pesticides, has a significant impact on the phylloplane’s microbial diversity. Yet the results on yeasts have received less attention, although the effects of fungicides on non-target yeasts have been documented (Cadez et al. 2010; Andreolli et al. 2021). Recently, Noel et al. (2022) demonstrated that foliar fungicides affect basidiomycetous yeast populations in the phylloplane of maize and soybean. However, data on the impact of crop management on phylloplane yeasts and the role of neighboring areas in maintaining microbial diversity are still in short supply.

The aim of this study is to identify and characterize basidiomycetous yeasts in the phylloplane of plants in typical Italian agroecosystems, such as vineyards, orchards and olive groves. Preliminary molecular fingerprinting was conducted to assess the level of intra-specific variability among isolates. Strains were tested for two important phenotypic traits based on their potential ability to adapt and survive in the phylloplane of agroecosystems, that is, the production of extracellular enzymes and the sensitivity to some common fungicides.

## Materials and methods

### Sampling and isolation of strains

Samples grown in different areas of some typical agroecosystems of Italy, such as vineyards (grapevine leaves and berries), orchards (e.g., olive trees or olive, apples and cherries) and surrounding areas (i.e., spontaneous herbaceous and horticultural plants), were collected during the period 2019–2023 (Table S1). The main geographical sampling area, where most of samples were collected, was plan and hilly lands of west of Veneto region (provinces of Verona − 45°26’N 10°59’E and Vicenza – 45°33’N 11°33’E), while few samplings were carried out in three areas (provinces of Trento − 46°04’N 11°07’E, Udine − 46°03’N 13°13’E and Taranto − 40°28’N 17°14’E) at different distance from the former. In all sampling areas, crops were treated with organic methods or not treated with synthetic fungicides or were abandoned. These areas had wooded or riparian zones or uncultivated fields adjacent the crop fields. Entire leaves or fruits were cut from the plant with ethanol-sterilized scissors, then collected into a sterile bag and transferred in laboratory for analysis. Portions of each sample were placed in sterile tube with an isotonic peptone solution (1 g/L peptone with 0.1 g/L polysorbate 80) that was incubated at 25 °C for 3 h with slow shaking to release microorganisms. Undiluted or diluted aliquots (100 µL) of suspension were plated on YPD agar (10 g/L yeast extract, 20 g/L peptone, 20 g/L glucose and 15 g/L agar) supplemented with 0.01% (w/v) chloramphenicol (Merck KGaA, Darmstadt, Germany) to inhibit bacterial growth. After 3–4 days of incubation at 25 °C, morphology of colonies and cells were analysed in order to select representative isolates of each sample, avoiding to collect clones. More than 100 isolates were selected and submitted to molecular identification.

## Species identification of isolates

The yeasts identification at species level was carried out through sequence analysis of the D1/D2 domains of 26 S rRNA gene. The DNA was extracted from 2 to 4 days old YPD broth culture using a Wizard^®^, Genomic DNA purification kit (Promega, Madison, WI). Total DNA were amplified by PCR using primers NL1/NL4 (Kurtzman and Robnett 1998). Amplicons were purified with a commercial kit (Nucleospin^®^ Gel and PCR Clean-up, Macherey-Nagel, Duren, Germany) and sequenced at Eurofins Genomics (Eurofins Genomics, Edersberg, Germany) using the same primers as for PCR reaction. A sequence similarity search was performed for species identification on type strain sequences deposited in NCBI-GenBank (https://blast.ncbi.nlm.nih.gov) and MycoBank database (http://mycobank.org), considering the highest score obtained in each pairwise alignment.

## Intraspecific differentiation by PCR fingerprinting

Molecular fingerprinting of strains belonging to the same species was carried out by PCR using primer (GTG)_5_ according to Lorenzini et al. (2023). Amplified products were visualized on agarose gel (1.5% w/v) stained with EuroSafe Nucleic Acid Stain (Euroclone spa, Milan, Italy) and acquisition of gel image was carried out by Gel Imaging Systems apparatus (Bio-Rad, Laboratories Inc., Hercules, CA). PCR reactions were performed in triplicate and fingerprinting analysis was carried out evaluating the reproducibility of each clearly visible band. The pattern obtained by manual selection of bands was numerically analysed with band-based dendrogram obtained using hierarchical clustering with Dice coefficient similarity and unweighted pair-group average as agglomeration method (statistical package XLSTAT 2018, Addinsoft SARL, Paris, France).

## Assays on activity of extracellular hydrolytic enzymes

All isolates were tested for the activity of six classes of extracellular hydrolytic enzymes (lipases, proteases, *β*-glucosidases, pectinases, cellulases and amylases). Assays were carried out in solid medium according to previous investigations (Brizzio et al. 2002; Martinez et al. 2016; González Flores et al. 2017). Lipolytic activity was detected on a medium containing polysorbate 20 (10 g/L polysorbate 20, 10 g/L peptone, 5 g/L NaCl, 0.1 g/CaCl_2_ and 10 g/L agar, pH 6.8). After incubation of 5 days the activity was detected as an opaque halo around the colony in clear medium (Brizzio et al. 2002). Proteolytic activity was verified on skim milk agar containing 10 g/L skim milk powder and 20 g/L agar (pH 6.6), and after 4–5 days of incubation, a clear zone around the colony in opaque medium indicated the positive reaction (Martinez et al. 2016). The *β*-glucosidases activity was assayed on medium containing esculin (3 g/L esculin, 2.5 g/L ammonium ferric citrate, 10 g/L yeast extract, 2 g/L dextrose, 20 g/L peptone, 10 g/L agar, pH 5). Strains showing this activity produced a dark brown halo around the colony after 1–3 days of incubation (González Flores et al. 2017). Pectinolytic activity was assayed on medium consisting of 10 g/L apple pectin, 2.0 g/L KH_2_PO_4_, 0.05 g/L CaCl_2_, 1.4 g/L of (NH_4_)_2_SO_4_, 0.2 g/L MgSO_4_, 1 mL solution containing 5 mg/L FeSO_4_, 1.6 mg/L MnSO_4_, 2 mg CoCl_2_ and 20 g/L agar. Plates were incubated for 2–4 days and the activity was detected by a clear yellow halo around the colony on purple-brown medium after Lugol’s iodine solution addition (Martinez et al. 2016). Cellulolytic activity was assayed on carboxymethylcellulose agar medium containing 2.0 g/L carboxymethylcellulose sodium salt, 2.0 g/L NaNO_3_, 1.0 g/L K_2_HPO_4_, 0.5 g/L MgSO_4_, 0.5 g/L KCl, 0.2 g/L peptone and 20 g/L agar. After incubation (2–4 days) positive reaction was observed after flooded the plates with Congo red solution (1 g/L) and subsequently destained with 1 M NaCl. The presence of cellulolytic activity was observed by a yellow halo around the colony (Martinez et al. 2016). Amylolytic activity was screened using starch agar medium containing 10 g/L soluble starch, 2 g/L yeast extract, 5 g/L peptone, 0.5 g/L MgSO_4_, 0.5 g/L NaCl, 0.15 g/L CaCl_2_ and 20 g/L agar (pH 6.0). After 3–4 days of incubation plates were flooded with Lugol’s iodine solution and positive reaction produced a pale yellow halo around the colony (Martinez et al. 2016). The temperature of plate incubation in all assays was 25 °C. Each isolate was tested in triplicate. Activities were visually evaluated and based on size of halo around the colony it was arbitrarily attributed an activity level as high (h), moderate (m), low (l) and absent (a).

## Assay on fungicidal sensitivity of isolates

All isolates were assayed for sensitivity to six commercial formulations of synthetic fungicides (Lidal^®^, Cantus^®^, Prolectus^®^, Tucana^®^, Carson^®^, Folpan^®^) used in Italy to control fungal pathogens of grapevines, fruit trees and horticultures (Table S2). Each fungicide was used separately and added in YPD agar after the sterilization at a concentration corresponding to the maximum dosage allowed in cultures above cited according to information reported in the commercial product (Lidal^®^ 3.75 µL/mL, Cantus ^®^ 1.20 mg/mL, Prolectus^®^ 50WG 1.0 mg/mL, Tucana^®^ 0.4 µL/mL, Carson^®^ 1.35 mg/mL and Folpan^®^ 2.0 mg/mL). Strains were inoculated on plates with 20 µL from culture grown 24–48 h in YPD broth, then plates were incubated at 25 °C. The growth of colonies was observed daily and after 4 days of incubation the level of sensitivity to each fungicide was assigned according to the colony growth (diameter, form and profile): resistant (r) when colony was like control; weakly sensitive (ws) when colony diameter was smaller than control (up to 50%); sensitive (s) when colony was very faint (the diameter was maximum 20% of the control) or very small colonies were observed; highly sensitive (hs) when the growth was totally absent. No intermedia growth between weakly sensitive and sensitive was observed. The assay was carried out in triplicate and three independent trials for each isolate were performed.

## Results

### Species identification and strain typing

On analysing 83 isolates, a total of 25 species belonging to 17 genera of subphyla Pucciniomycotina, Agaricomycotina and Ustilaginomycotina were identified (Table 1). The most frequent genera were *Filobasidium*, represented by the species *F. magnum*, *F. oeirense* and *F. wieringae*, and *Rhodotorula* in the form of *R. graminis*, *R. mucilaginosa* and *R. dairenensis*. Several isolates belonging to genera such as *Sporobolomyces*, *Sporidiobolus*, *Rhodosporidiobolus*, *Curvibasidium*, *Vishniacozyma*, *Papiliotrema* and *Kwoniella* were also identified. The genera *Cystobasidium*, *Symmetrospora*, *Buckleyzyma*, *Naganishia*, *Trichosporon*, *Hannaella*, *Pseudozyma* and *Moniliella* were represented by a single isolate.

**Table 1:** Species identification of 83 isolates by D1/D2 domain sequence similarity searches on nucleotide sequence databases considering the highest score obtained in each pairwise alignment

Strain	Species	Accession Number	Most similar type strain	Accession Number	Similarity %
**Pucciniomycotina**					
V-V16	*Rhodotorula graminis*	OR717069	*Rhodotorula graminis* CBS 2826^T^	NG_068963	100
V-V23	*Rhodotorula graminis*	OR717070	*Rhodotorula graminis* CBS 2826^T^	NG_068963	100
V-V24	*Rhodotorula graminis*	OR717071	*Rhodotorula graminis* CBS 2826^T^	NG_068963	100
V-V26	*Rhodotorula graminis*	OR717072	*Rhodotorula graminis* CBS 2826^T^	NG_068963	100
V-V27	*Rhodotorula graminis*	OR717073	*Rhodotorula graminis* CBS 2826^T^	NG_068963	100
V-V28	*Rhodotorula graminis*	OR717074	*Rhodotorula graminis* CBS 2826^T^	NG_068963	100
V-T5	*Rhodotorula graminis*	OR717075	*Rhodotorula graminis* CBS 2826^T^	NG_068963	100
V-T8	*Rhodotorula graminis*	OR717076	*Rhodotorula graminis* CBS 2826^T^	NG_068963	100
V-T13	*Rhodotorula graminis*	OR717077	*Rhodotorula graminis* CBS 2826^T^	NG_068963	100
V-U4	*Rhodotorula graminis*	OR717078	*Rhodotorula graminis* CBS 2826^T^	NG_068963	99.79
O-V30	*Rhodotorula graminis*	OR717079	*Rhodotorula graminis* CBS 2826^T^	NG_068963	100
O-V32	*Rhodotorula graminis*	OR717080	*Rhodotorula graminis* CBS 2826^T^	NG_068963	100
O-V38	*Rhodotorula graminis*	OR717081	*Rhodotorula graminis* CBS 2826^T^	NG_068963	100
S-V43	*Rhodotorula graminis*	OR717082	*Rhodotorula graminis* CBS 2826^T^	NG_068963	99.66
S-T16	*Rhodotorula graminis*	OR717083	*Rhodotorula graminis* CBS 2826^T^	NG_068963	100
S-Ta4	*Rhodotorula graminis*	OR717084	*Rhodotorula graminis* CBS 2826^T^	NG_068963	99.68
S-Ta10	*Rhodotorula graminis*	OR717085	*Rhodotorula graminis* CBS 2826^T^	NG_068963	100
V-V15	*Rhodotorula mucilaginosa*	OR717086	*Rhodotorula mucilaginosa* CBS 316^T^	NG_055716	100
O-V40	*Rhodotorula mucilaginosa*	OR717087	*Rhodotorula mucilaginosa* CBS 316^T^	NG_055716	99.81
V-V20	*Rhodotorula dairenensis*	OR717088	*Rhodotorula dairenensis* CBS 4406^T^	NG_057644	99.58
O-V29	*Rhodotorula dairenensis*	OR717089	*Rhodotorula dairenensis* CBS 4406^T^	NG_057644	99.56
V-T2	*Sporobolomyces sucorum*	OR717090	*Sporobolomyces sucorum* CBS 15,628 ^T^	MG478490	100
V-T4	*Sporobolomyces sucorum*	OR717091	*Sporobolomyces sucorum* CBS 15,628 ^T^	MG478490	100
V-T11	*Sporobolomyces sucorum*	OR717092	*Sporobolomyces sucorum* CBS 15,628 ^T^	MG478490	100
O-V37	*Sporobolomyces sucorum*	OR717093	*Sporobolomyces sucorum* CBS 15,628^T^	MG478490	100
O-V41	*Sporobolomyces sucorum*	OR717094	*Sporobolomyces sucorum* CBS 15,628^T^	MG478490	100
S-T15	*Sporobolomyces sucorum*	OR717095	*Sporobolomyces sucorum* CBS 15,628^T^	MG478490	100
V-V3	*Sporidiobolus metaroseus*	OR717096	*Sporidiobolus metaroseus* CBS 7683^T^	EU003461	99.78
V-T6	*Sporidiobolus metaroseus*	OR717097	*Sporidiobolus metaroseus* CBS 7683^T^	EU003461	99.80
V-U2	*Sporidiobolus metaroseus*	OR717098	*Sporidiobolus metaroseus* CBS 7683^T^	EU003461	99.76
S-V42	*Sporidiobolus metaroseus*	OR717099	*Sporidiobolus metaroseus* CBS 7683^T^	EU003461	99.80
S-Ta1	*Sporidiobolus metaroseus*	OR717100	*Sporidiobolus metaroseus* CBS 7683^T^	EU003461	99.76
S-Ta6	*Sporidiobolus metaroseus*	OR717101	*Sporidiobolus metaroseus* CBS 7683^T^	EU003461	99.75
V-V5	*Rhodosporidiobolus fluvialis*	OR717102	*Rhodosporidiobolus fluvialis* CBS 6568 ^T^	KY108963	100
S-V45	*Rhodosporidiobolus colostri*	OR699280	*Rhodosporidiobolus colostri* CBS 348^T^	NG_070510	100
V-V12	*Curvibasidium pallidicorallinum*	OR717103	*Curvibasidium pallidicorallinum* CBS 9091^T^	KY107299	99.78
V-V14	*Curvibasidium pallidicorallinum*	OR717104	*Curvibasidium pallidicorallinum* CBS 9091^T^	KY107299	99.51
V-V17	*Curvibasidium pallidicorallinum*	OR717105	*Curvibasidium pallidicorallinum* CBS 9091^T^	KY107299	100
O-V35	*Curvibasidium pallidicorallinum*	OR717106	*Curvibasidium pallidicorallinum* CBS 9091^T^	KY107299	99.51
S-V47	*Curvibasidium pallidicorallinum*	OR717107	*Curvibasidium pallidicorallinum* CBS 9091^T^	KY107299	100
S-V51	*Curvibasidium cygneicollum*	OR717108	*Curvibasidium cygneicollum* CBS 4551^T^	KY107291	100
S-Ta5	*Cystobasidium slooffiae*	OR717109	*Cystobasidium slooffiae* CBS 5706^T^	NG_059008	99.70
V-V10	*Symmetrospora coprosmae*	OR717110	*Symmetrospora coprosmae* CBS 7899^T^	NG_067795	100
S-V49	*Buckleyzyma salicina*	OR717111	*Buckleyzyma salicina* CBS 6983^T^	NG_058619	100
**Agaricomycotina**					
V-V1	*Filobasidium magnum*	OR717112	*Filobasidium magnum* CBS 140^T^	NG_069409	99.81
V-V4	*Filobasidium magnum*	OR717113	*Filobasidium magnum* CBS 140^T^	NG_069409	100
V-V9	*Filobasidium magnum*	OR717114	*Filobasidium magnum* CBS 140^T^	NG_069409	100
V-V13	*Filobasidium magnum*	OR717115	*Filobasidium magnum* CBS 140^T^	NG_069409	100
V-V22	*Filobasidium magnum*	OR717116	*Filobasidium magnum* CBS 140^T^	NG_069409	100
V-T3	*Filobasidium magnum*	OR717117	*Filobasidium magnum* CBS 140^T^	NG_069409	100
V-T10	*Filobasidium magnum*	OR717118	*Filobasidium magnum* CBS 140^T^	NG_069409	100
O-V31	*Filobasidium magnum*	OR717119	*Filobasidium magnum* CBS 140^T^	NG_069409	99.79
O-V36	*Filobasidium magnum*	OR717120	*Filobasidium magnum* CBS 140^T^	NG_069409	100
S-T14	*Filobasidium magnum*	OR717121	*Filobasidium magnum* CBS 140^T^	NG_069409	99.82
S-Ta3	*Filobasidium magnum*	OR717122	*Filobasidium magnum* CBS 140^T^	NG_069409	99.79
V-V2	*Filobasidium oeirense*	OR717123	*Filobasidium oeirense* CBS 8681^T^	NG_070508	100
V-V7	*Filobasidium oeirense*	OR717124	*Filobasidium oeirense* CBS 8681^T^	NG_070508	100
S-V46	*Filobasidium oeirense*	OR717125	*Filobasidium oeirense* CBS 8681^T^	NG_070508	100
V-V25	*Filobasidium wieringae*	OR717126	*Filobasidium wieringae* CBS 1937^T^	NG_067314	99.81
V-T1	*Filobasidium wieringae*	OR717127	*Filobasidium wieringae* CBS 1937^T^	NG_067314	99.79
V-T7	*Filobasidium wieringae*	OR717128	*Filobasidium wieringae* CBS 1937^T^	NG_067314	100
V-U1	*Filobasidium wieringae*	OR717129	*Filobasidium wieringae* CBS 1937^T^	NG_067314	100
O-V42	*Filobasidium wieringae*	OR717130	*Filobasidium wieringae* CBS 1937^T^	NG_067314	100
S-V50	*Filobasidium wieringae*	OR717131	*Filobasidium wieringae* CBS 1937^T^	NG_067314	100
V-V21	*Naganishia diffluens*	OR717132	*Naganishia diffluens* CBS 160^T^	NG_058351	99.40
V-T12	*Vishniacozyma carnescens*	OR717133	*Vishniacozyma carnescens* CBS 973^T^	NG_058430	99.81
S-Ta2	*Vishniacozyma carnescens*	OR717134	*Vishniacozyma carnescens* CBS 973^T^	NG_058430	100
S-Ta7	*Vishniacozyma carnescens*	OR717135	*Vishniacozyma carnescens* CBS 973^T^	NG_058430	99.75
S-Ta8	*Vishniacozyma carnescens*	OR717136	*Vishniacozyma carnescens* CBS 973^T^	NG_058430	99.75
V-V19	*Trichosporon asahii*	OR717137	*Trichosporon ahsaii* CBS 2479^T^	NG_055732	100
V-V6	*Papiliotrema flavescens*	OR717138	*Papiliotrema flavescens* CBS 942^T^	AB035042	100
V-U3	*Papiliotrema flavescens*	OR717139	*Papiliotrema flavescens* CBS 942^T^	AB035042	100
O-V39	*Papiliotrema flavescens*	OR717140	*Papiliotrema flavescens* CBS 942^T^	AB035042	100
S-V44	*Papiliotrema flavescens*	OR717141	*Papiliotrema flavescens* CBS 942^T^	AB035042	100
S-V48	*Papiliotrema flavescens*	OR717142	*Papiliotrema flavescens* CBS 942^T^	AB035042	99.80
S-V52	*Papiliotrema flavescens*	OR717143	*Papiliotrema flavescens* CBS 942^T^	AB035042	100
S-Ta9	*Papiliotrema aurea*	OR717144	*Papiliotrema aurea* CBS 318^T^	NG_148937	99.77
O-V33	*Kwoniella mangroviensis*	OR717145	*Kwoniella mangroviensis* CBS 8507^T^	KY108202	99.45
O-V34	*Kwoniella mangroviensis*	OR717146	*Kwoniella mangroviensis* CBS 8507^T^	KY108202	99.80
V-T9	*Kwoniella pini*	OR717147	*Kwoniella pini* CBS 10,737^T^	KY10820	99.40
V-V11	*Hannaella sinensis*	OR717148	*Hannaella sinensis* CBS 7238^T^	NG_042362	100
	**Ustilaginomycotina**				
V-V18	*Pseudozyma prolifica*	OR717149	*Pseudozyma prolifica* CBS 319.87^T^	MH873769	100
V-V8	*Moniliella megachiliensis*	OR717150	*Moniliella megachiliensis* CBS 190.92^T^	NG_070600	99.17

Species like *R. graminis*, *F. magnum*, *F. wieringae*, *S. sucorum*, *C. pallidicorallinum*, *V. carnescens* and *P. flavescens* were frequently found, both in crop plants and spontaneous plants sampled in the areas surrounding cultivated fields. Similarly, strains of unique species (e.g., *Rh. fluvialis*, *Rh. colostri*, *Cy. slooffiae* and *Sy. coprosmae*) were isolated from the phylloplane of both cultivated and spontaneous plants.

Isolates belonging to the same species were analysed by (GTG)_5_-PCR fingerprinting to acquire preliminary information on intra-specific molecular variability. Fig. S1 shows dendrograms obtained by clustering analysis, that displayed similarities within isolates of the same genus, including *Sporobolomyces*/*Sporidiobolus* genera. *Rhodotorula graminis* isolates displayed similarity ranging from 68 to 100% and were grouped in two main clusters, A and B, of 8 and 9 isolates, respectively (Fig. S1A). Different fingerprinting profiles were also observed within the two isolates of *R. dairenensis* and *R. mucilaginosa* (Fig. S1A). Isolates of *Sp. metaroseus* showed different fingerprinting profiles with similarity ranging from 61 to 83% while, among *S. sucorum* isolates, three of them (V-T4, O-V37 and O-V41) had identical profiles (100%). The least similar was V-T11 (69%) (Fig. S1B). Similarity of 52 and 89% was found among *C. pallidicorallinum* isolates, while *C. cygneicollum* S-51 displayed a very different profile (28% similarity) (Fig. S1C). *Filobasidium* isolates were found in three main clusters A, B and C (Fig. S1D). Cluster C contained only *F. wieringae* isolates with similarity ranging from 58 to 100%, while *F. magnum* isolates, which displayed high variability (92 − 39% similarity) were contained in clusters A and B. This last cluster also had three *F. oeirense* isolates, two with identical profiles (100%) and a third that displayed a similarity of 92%. Heterogenicity was observed in all four *V. carnescens* isolates, which displayed maximum similarity of 85% (Fig. S1E), as well as in *P. flavescens* isolates, all of which were all discriminated (73–96%), except isolates *P. flavescens* V-U3 and S-V48, which had similarity of 100% (Fig. S1F). The profile of *P. aurea* S-Ta9 was very different to all *P. flavescens* isolates (52% similarity). The (GTG)_5_ PCR fingerprinting profiles of two *K. mangroviensis* isolates were identical (100%) (data not shown).

## Extracellular enzyme activity assays

**Fig. 1:**
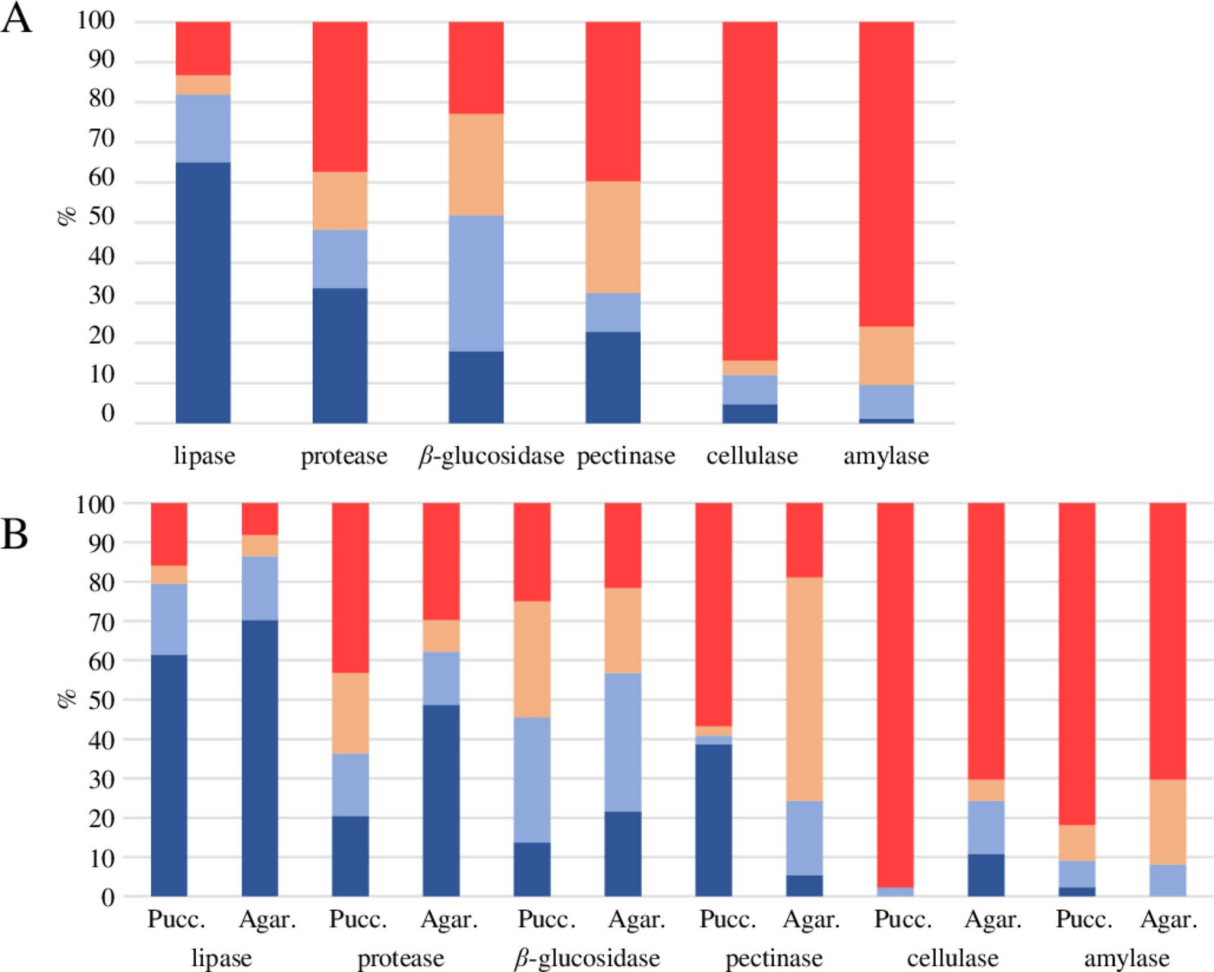
Percentage of isolates that showed activity of six extracellular hydrolytic enzymes on a total of 83 yeasts (100%) (**A**), and 81 isolates grouped in Pucciniomycotina (44 isolates, 53%) and Agaricomycotina (37 isolates, 44%) (**B**). Levels of activity: high, blue; moderate, light blue; low, light red; absent, red

Most isolates displayed lipolytic (87%), proteolytic (63%), *β*-glucosidases (77%) and pectinolytic (60%) activities, while only 16% and 24%, respectively, displayed cellulolytic and amylolytic activity (Fig. 1A). High and moderate lipolytic activity was observed in 82% isolates. Substantial differences in their protealytic, pectinolytic and cellulolytic activities were observed between the two subphyla Pucciniomycotina and Agaricomycotina (44 and 37 isolates, respectively) (Fig. 1B).

These enzymatic activities were detected at high or moderate levels of 36, 41 and 2% in Pucciniomycotina strains and 62, 24 and 24% in Agaricomycotina strains, respectively. Moreover, significant inter- and intra-specific heterogenicity was observed (Table 2).

**Table 2:** Activity of six extracellular hydrolytic enzymes of 83 basidiomycetous isolates from phylloplane grouped for species, tested by plate assay

	*n*. isolates	lipases	proteases	β-glucosidase	pectinase	cellulase	amylase
*R. graminis*	17	17 (h^1^)	7 (h), 7 (m), 3 (l)	10 (m), 5 (l), 2 (a)	1 (h), 16 (a)	17 (a)	2 (l), 15 (a)
*R. mucilaginosa*	2	2 (m)	2 (a)	2 (a)	2 (a)	2 (a)	2 (a)
*R. dairenensis*	2	2 (l)	2 (a)	2 (a)	2 (a)	2 (a)	2 (a)
*S. sucorum*	6	3 (h), 3 (m)	4 (l), 2 (a)	6 (h)	6 (h)	6 (a)	6 (a)
*Sp. metaroseus*	6	6 (h)	2 (h), 2(l), 2(a)	5 (h), 1 (l)	5 (h), 1(l)	6 (a)	6 (a)
*Rh. fluvialis*	1	1 (m)	1 (a)	1 (h)	1 (a)	1 (a)	1 (a)
*Rh. colostri*	1	1 (a)	1 (a)	1 (l)	1 (a)	1 (a)	1 (a)
*C. pallidicorallinum*	5	5 (a)	5 (a)	3 (m), 2 (l)	5 (h)	5 (a)	1 (h), 3 (m), 1 (l)
*C. cygneicollum*	1	1 (a)	1 (a)	1 (l)	1 (m)	1 (a)	1 (1)
*Cy. slooffiae*	1	1 (h)	1 (a)	1 (l)	1 (a)	1 (a)	1 (a)
*Sy. coprosmae*	1	1 (m)	1 (a)	1 (h)	1 (a)	1 (m)	1 (a)
*B. salicina*	1	1 (m)	1 (a)	1 (h)	1 (a)	1 (a)	1 (a)
*F. magnum*	11	10 (h), 1 (l)	8 (h), 2 (m), 1 (l)	2 (h), 6 (m), 1 (l), 2 (a)	3 (m), 7 (l), 1 (a)	1 (m), 1 (l), 9 (a)	2 (m), 2 (l), 7 (a)
*F. oeirense*	3	2 (h), 1 (m)	3 (a)	3 (m)	3 (l)	3 (h)	3 (a)
*F. wieringae*	6	6 (h)	4 (h), 2 (m)	4 (h), 2 (m)	2 (m), 4 (l)	1 (h), 5 (a)	1 (m), 1 (l), 4 (a)
*N. diffluens*	1	1 (h)	1 (l)	1 (l)	1 (a)	1 (a)	1 (a)
*V. carnescens*	4	2 (m), 2 (a)	4 (a)	2 (l), 2(a)	1 (l), 3 (a)	3 (m), 1 (a)	2 (m), 2(a)
*T. asahii*	1	1 (m)	1 (a)	1 (h)	1 (a)	1 (a)	1 (a)
*P. flavescens*	6	6 (h)	5 (h), 1 (m)	1 (l), 5 (a)	1 (h), 5(l)	6 (a)	1 (l), 5 (a)
*P. aurea*	1	1 (h)	1 (h)	1 (l)	1 (l)	1 (a)	1 (a)
*K. mangroviensis*	2	1 (l), 1 (a)	1 (l), 1 (a)	2 (l)	1(h), 1 (m)	1 (m), 1 (l)	1 (l), 1 (a)
*K. pini*	1	1 (m)	1 (a)	1 (a)	1 (m)	1 (a)	1 (l)
*H. sinensis*	1	1 (m)	1 (a)	1 (h)	1 (a)	1 (a)	1 (a)
*Pr. prolifica*	1	1 (h)	1 (h)	1 (h)	1 (l)	1 (l)	1 (m)
*M. megachiliensis*	1	1 (a)	1 (a)	1 (h)	1 (a)	1 (a)	1 (a)

^1^ Levels of activity: h, high; m, moderate; l, low; a, absent

In Pucciniomycotina, strains of *R. graminis*, *S. sucorum* and *C. pallidicorallinum* displayed different levels of activity in all enzymes, except cellulase. In particular, isolates of *R. graminis* and *Sp. metaroseus* were observed that displayed both high and zero activity for pectinase and proteases, respectively. Moreover, lipolytic activity was absent in all *C. pallidicorallinum* strains and only one isolate of this species (V-V12) displayed elevated amylolytic activity among all the 83 isolates tested. In Agaricomycotina, *F. magnum* strains displayed great variability in all six enzymes. Strains of the same species had both high or moderate activity and low or zero activity of pectinase, cellulase and amylase (*F. wieringae*), pectinase (*P. flavescens*), and lipase, cellulase and amylase (*V. carnescens*).

Three isolates, *F. magnum* O-V31, *K. mangroviensis* O-V33 and *Pr. prolifica* V-V18, displayed all six enzymatic activities, while 7 isolates presented only one activity (lipase for *R. mucilaginosa* and *R. dairenensis* strains, *β*-glucosidase for *Rh. colostri* S-V45 and *M. megachiliensis* V-V8, cellulase for *V. carnescens* S-Ta8) (Table S3).

### Fungicide sensitivity assays

**Fig. 2:**
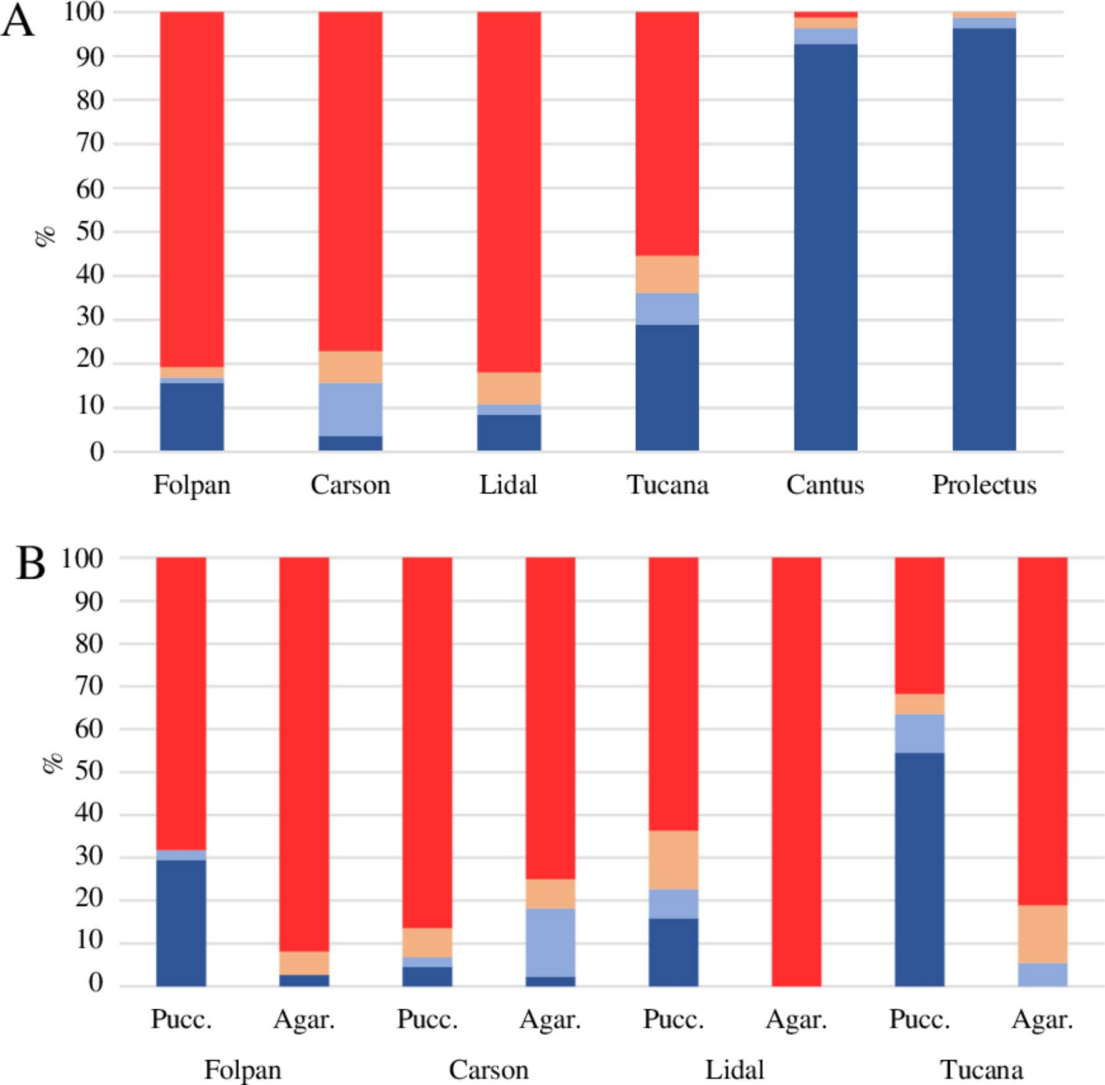
Percentage of isolates that showed sensitivity to six commercial formulation of synthetic fungicides on a total of 83 yeasts (100%) (**A**), and sensitivity to four fungicides on 81 isolates grouped in Pucciniomycotina (44 isolates, 53%) and Agaricomycotina (37 isolates, 44%) (**B**). Level of sensitivity: resistant, blue; weakly sensitive, light blue; sensitive, light red; highly sensitive, red

Differences were observed in the growth of the isolates depending on the fungicides used. Most isolates were sensitive (evaluated as sensitive and highly sensitive in the plate assay) to Folpan^®^ (83%), Tucana^®^ (64%), Carson^®^ (84%) and Lidal^®^ (89%). Conversely, inhibitory effects of Prolectus^®^ and Cantus^®^ were observed in only a few isolates (Fig. 2A). As regards the difference between Pucciniomycotina and Agaricomycotina, the former displayed more isolates that were resistant or weakly sensitive to Folpan^®^ (32% vs. 3%), Tucana^®^ (64% vs. 5%) and Lidal^®^ (23% vs. 0%) (Fig. 2B). On the other hand, Agaricomycotina displayed more isolates that were resistant or weakly sensitive to Carson^®^ than those belonging to Pucciniomycotina subphylum (21% vs. 7%).

Intraspecific variability in fungicide sensitivity was observed (Table 3). Within the same species, isolates were found that were both resistant or weakly sensitive and sensitive or highly sensitive to a certain fungicide. For example, isolates that were both resistant and highly sensitive to Folpan^®^, Carson^®^ and Lidal^®^ were found in *R. graminis*, to Tucana^®^ in *Sp. metaroseus*, or to Folpan^®^ in *F. magnum*. Some isolates, such as *R. graminis* V-V28 and S-T16, were resistant or weakly sensitive to all five fungicides. Conversely, *B. salicina* S-V49 and *S. coprosmae* V-V10 were highly sensitive or sensitive to all fungicides (Table S4).

**Table 3:** Sensitivity of 83 basidiomycetous isolates grouped by species to six commercial formulations of fungicides (Folpan^®^ 2.0 mg/mL, Carson^®^ 1.35 mg/mL, Lidal^®^ 3.75 µL/mL, Tucana^®^ 0.4 µL/mL, Cantus^®^ 1.20 mg/mL, and Prolectus^®^ 50WG 1.0 mg/mL) tested by plate assay

species	*n*. isolates	Folpan^®^	Carson^®^	Lidal^®^	Tucana^®^	Cantus^®^	Prolectus^®^
*R. graminis*	17	8 (r^1^), 9 (hs)	2 (r), 1 (ws), 3 (s), 11 (hs)	3 (r), 1 (ws), 3 (s), 10 (hs)	15 (r), 2 (ws)	17 (r)	17 (r)
*R. mucilaginosa*	2	2 (hs)	2 (hs)	2 (s)	2 (r)	2 (r)	2 (r)
*R. dairenensis*	2	2 (hs)	2 (hs)	2 (hs)	2 (r)	2 (r)	2 (r)
*S. sucorum*	6	5 (r), 1 (ws)	6 (hs)	4 (r), 2 (ws)	4 (r), 1 (ws), 1 (s)	6 (r)	6 (r)
*Sp. metaroseus*	6	6 (hs)	6 (hs)	1 (s), 5 (hs)	1 (r), 5 (hs)	6 (r)	6 (r)
*Rh. fluvialis*	1	1 (hs)	1 (hs)	1 (hs)	1 (hs)	1 (r)	1 (r)
*Rh. colostri*	1	1 (hs)	1 (hs)	1 (hs)	1 (hs)	1 (r)	1 (r)
*C. pallidicorallinum*	5	5 (hs)	5 (hs)	5 (hs)	1 (ws), 1 (s), 3 (hs)	5 (r)	5 (r)
*C. cygneicollum*	1	1 (hs)	1 (hs)	1 (hs)	1 (hs)	1 (r)	1 (r)
*Cy. slooffiae*	1	1 (hs)	1 (hs)	1 (hs)	1 (hs)	1 (r)	1 (r)
*Sy. coprosmae*	1	1 (hs)	1 (hs)	1 (hs)	1 (hs)	1 (hs)	1 (ws)
*B. salicina*	1	1 (hs)	1 (hs)	1 (hs)	1 (hs)	1 (hs)	1 (r)
*F. magnum*	11	1 (r), 1 (s), 9 (hs)	2 (s), 9 (hs)	11 (hs)	1 (ws), 1 (s), 9 (hs)	11 (r)	11 (r)
*F. oeirense*	3	3 (hs)	3 (hs)	3 (hs)	3 (hs)	2 (ws), 1 (s)	2 (r), 1 (ws)
*F. wieringae*	6	6 (hs)	6 (hs)	6 (hs)	6 (hs)	5 (r), 1 (ws)	5 (r), 1(s)
*N. diffluens*	1	1 (hs)	1 (ws)	1 (hs)	1 (hs)	1 (r)	1 (r)
*V. carnescens*	4	4 (hs)	4 (hs)	4 (hs)	1 (s), 3(hs)	4 (r)	4 (r)
*T. asahii*	1	1 (s)	1 (r)	1 (hs)	1 (s)	1 (r)	1 (r)
*P. flavescens*	6	6 (hs)	5 (ws), 1 (s)	6 (hs)	4 (r), 2 (s)	6 (r)	6 (r)
*P. aurea*	1	1 (hs)	1 (ws)	1 (hs)	1 (hs)	1 (r)	1 (r)
*K. mangroviensis*	2	2 (hs)	2 (hs)	2 (hs)	1 (ws), 1 (hs)	2 (r)	2 (r)
*K. pini*	1	1 (hs)	1 (ws)	1 (hs)	1 (hs)	1 (r)	1 (r)
*H. sinensis*	1	1 (hs)	1 (ws)	1 (hs)	1 (hs)	1 (s)	1 (r)
*Pr. prolifica*	1	1 (hs)	1 (hs)	1 (hs)	1 (hs)	1 (r)	1 (r)
*M. megachiliensis*	1	1 (hs)	1 (hs)	1 (hs)	1 (r)	1 (r)	1 (r)

^1^ level of sensitivity: r, resistant; ws, weakly sensitive; s, sensitive; hs, highly sensitive

## Discussion

All 25 species identified in this study had already been detected in the phylloplane of different plants in various habitats (Kurtzman et al. 2011). Most of these species are saprophytic yeasts and those belonging to the genera *Rhodotorula*, *Sporobolomyces*, *Sporidiobolus*, *Curvibasidium*, *Filobasidium*, *Papiliotrema* and *Vishniacozyma* are ubiquitous (Cray et al. 2013; Kemler et al. 2017; Li et al. 2020; Andreolli et al. 2021; Gouka et al. 2022). Moreover, some of these basidiomycetous yeasts can easily be isolated from extreme natural environments (e.g., polar habitats, seawater) (Buzzini et al. 2018). The prevalence of a few yeast species from different areas of the agroecosystems analysed in this study confirms that they have great capacity for adaptation and proliferation in these habitats. Insects have been shown to be important vectors that contribute to maintaining and spreading yeast populations in natural environments (Valentini et al. 2022). The frequent isolation of some species, like *R. graminis*, *S. sucorum*, *Sp. metaroseus*, *F. magnum*, *F. wieringae* and *C. pallidicorallinum*, both from crops (e.g., grapevine, olive tree) and neighbouring spontaneous plants (e.g., pokeweed, rosehip, butcher’s broom), is probably also due to the role of insects that visit the phylloplanes of both. Several species identified in this work such as *Sp. metaroseus*, *R. mucilaginosa*, *F. magnum*, *N. diffluens*, *V. carnescens*, *T. asahii*, and *P. flavescens*, have been isolated from flowers of fruit plants in Egypt (Moubasher et al. 2018). In addition, the occurrence of basidiomycetous yeasts in flowers can be also associated with birds. Mittelbach et al. (2015) isolated *V. carnescens*, *F. magnum*, *F. oeirense*, *P. aurea*, and *P. flavescens* from ornithophilous flowers. Our recovery of isolates belonging to these species on spontaneous plants is therefore evidence that basidiomycetous yeasts regularly inhabit flowers.

The intraspecific heterogeneity, revealed by strain genotype analysis, suggests that no relationship exists between genotype and geographical origin. This lack of correlation has been reported previously in different ecological studies on the structure of phylloplane yeast populations (Maganti et al. 2012; Dhami et al. 2018). On the other hand, ecological factors such as geographic barriers, insect vectors and host plants may affect the distribution of yeasts in their natural habitat and severely limit their diffusion (Yurkov 2017). It is possible that the lack of geographic and climate barriers in the common Italian agroecosystems (Costantini et al. 2013) may favor the dispersal of the genotypes of those yeasts most adapted to these habitats. Further studies with a greater number of samples and more extensive DNA fingerprinting analysis are required to confirm the supposition advanced by this work.

Data on enzymatic activities indicate that agroecosystems can harbour significant biochemical diversity within yeast populations, a phenomenon also observed in wild habitats, including extreme ones (Carrasco et al. 2012; Vidya et al. 2022). The dominance of lipolytic yeasts is consistent with the need of phylloplane yeasts to use the fatty acids contained in the plant cuticle, as previously reported (Molnárová et al. 2014; Ueda et al. 2015). Similarly, the prevalence of isolates with proteolytic, *β*-glucosidases and pectinolytic activity can be explained since their production allows the survival of these yeasts by using plant exudates and guttation droplets as nutrients. While the food industry is extremely interested in the use of pectinolytic and cellulolytic yeasts and also for cellulosic biomass hydrolysis, yeasts have rarely been examined for these properties (Haile and Ayele 2022; Sohail et al. 2022). The strains in this study with high pectinolytic and/or cellulolytic activity (e.g., *R. graminis* V-V26, *F. oeirense* V-V7, *F. wieringae* O-V42 and *K. mangroviensis* O-V33) therefore merit further investigation.

In terms of the fungicidal sensitivity of isolates, this study provides important information on the possible impact of synthetic fungicides on phylloplane yeasts in agroecosystems. The high percentage of yeasts sensitive to fungicides commonly used to control powdery and downy mildew (Folpan^®^, Tucana^®^, Carson^®^ and Lidal^®^) - serious pathogens of grapevines, fruit trees and other crops - suggests that basidiomycetous yeast populations may be undergoing a significative selective process in fields treated with these compounds. A reduction of the diversity of phylloplane yeasts due to the detrimental effects of fungicides has previously been described (Buck and Burpee 2002; Walter et al. 2007; Noel et al. 2022). Specifically, the inhibitory effects of triazoles, a fungicide class to which tetraconazole (Lidal^®^) belongs, were observed in some yeasts but not in others, due to the different relative sensitivity to fungicide (Buck and Burpee 2002; Walter et al. 2007). Our results showed that a few strains of *R. graminis* and *S. sucorum* are resistant to Lidal^®^. Noel et al. (2022) observed a decrease in the abundance of Tremellomycetes yeasts after foliar application of pyraclostrobin (Tucana^®^) and trifloxystrobin, which are QoI fungicides. These results agree with the high frequency of the agaricomycotina strains (all belonging to Tremellomycetes) sensitive to Tucana^®^ assayed in our study. The negative impact on yeasts during the fermentation of the grape must containing residues of pyraclostrobin and folpet has been previously documented (Zara et al. 2011; Russo et al. 2019). Furthermore, fungicidal treatments may lead to a reduction of non-target yeasts that have beneficial effects on plants (Andreolli et al. 2021). As well as differences between Pucciniomycotina and Agaricomycotina in their sensitivity to Folpan^®^, Tucana^®^, Carson^®^ and Lidal^®^, the screening involved in this study highlighted individual variability within the same species. This strain heterogeneity was also observed by Kosel et al. (2019), who assayed several species of yeasts from grape surfaces, and also reported that fungicide tolerance was a feature retained within the yeast’s genera. In our study, some phenotypes with rare traits of resistance, such as *R. graminis* V-V28 and S-Ta4 to Carson^®^, or *Sp. metaroseus* V-U2 to Tucana^®^ or *F. magnum* O-V31 to Folpan^®^, could prevail under selective pressure under these fungicides. Therefore, more yeasts can be expected to be isolated with these phenotypes in agricultural areas where crops are treated with these pesticides. The rapid development of fungicidal resistance in phytopathogenic fungi and yeasts that are pathogenic for humans is well-known and has been thoroughly investigated (Hahn 2014; Lee et al. 2020).

## Conclusions

The culturable approach of this study furnished data that contributed to the knowledge of the structure of yeast populations in agroecosystems. Unlike unculturable methods, it did not permit the in-depth exploration of the taxonomical composition of yeast microbiota. However, the survey of isolates revealed genetic and phenotypic heterogeneity that provides useful information on their potential ability to adapt to the environmental conditions that prevail in these habitats and the possible role played by natural areas adjacent to cultivated fields in maintaining yeast diversity. Furthermore, the culturable approach used in this study made it possible to contextually isolate and select strains that may be used in the industrial production of enzymes. Further investigations are clearly required to more fully understand the impact of traditional agricultural practices on the phylloplane fungal communities of agroecosystems.

## Electronic supplementary material

Below is the link to the electronic supplementary material.

Supplementary Material 1

## Data Availability

No datasets were generated or analysed during the current study.
