# Supplementary material for: Assessing variability among culturable phylloplane basidiomycetous yeasts from Italian agroecosystems
Source: World J Microbiol Biotechnol. 2024 Oct 3;40(11):335. doi: 10.1007/s11274-024-04147-z (PMC11446951; doi:10.1007/s11274-024-04147-z)
Supplement: Supplementary file 1 — Supplementary Material 1 [file 11274_2024_4147_MOESM1_ESM.pdf]

SUPPLEMETARY MATERIALS

Ferluga et al. "Assessing variability among culturable phylloplane basidiomycetous yeasts from Italian agroecosystems"

Table S1. List of 83 yeast isolates, source (species and part of plant used for the isolation) and location (administrative province).

Isolate	Source		Location
V-V1[1]	*Vitis vinifera*	leaf	Verona/Vicenza
V-V2	*Vitis vinifera*	leaf	Verona/Vicenza
V-V3	*Vitis vinifera*	leaf	Verona/Vicenza
V-V4	*Vitis vinifera*	leaf	Verona/Vicenza
V-V5	*Vitis vinifera*	leaf	Verona/Vicenza
V-V6	*Vitis vinifera*	leaf	Verona/Vicenza
V-V7	*Vitis vinifera*	leaf	Verona/Vicenza
V-V8	*Vitis vinifera*	leaf	Verona/Vicenza
V-V9	*Vitis vinifera*	leaf	Verona/Vicenza
V-V10	*Vitis vinifera*	leaf	Verona/Vicenza
V-V11	*Vitis vinifera*	leaf	Verona/Vicenza
V-V12	*Vitis vinifera*	leaf	Verona/Vicenza
V-V13	*Vitis vinifera*	leaf	Verona/Vicenza
V-V14	*Vitis vinifera*	leaf	Verona/Vicenza
V-V15	*Vitis vinifera*	leaf	Verona/Vicenza
V-V16	*Vitis vinifera*	leaf	Verona/Vicenza
V-V17	*Vitis vinifera*	leaf	Verona/Vicenza
V-V18	*Vitis vinifera*	leaf	Verona/Vicenza
V-V19	*Vitis* sp.	leaf	Verona/Vicenza
V-V20	*Vitis vinifera*	fruit	Verona/Vicenza
V-V21	*Vitis vinifera*	fruit	Verona/Vicenza
V-V22	*Vitis vinifera*	fruit	Verona/Vicenza
V-V23	*Vitis vinifera*	fruit	Verona/Vicenza
V-V24	*Vitis vinifera*	fruit	Verona/Vicenza
V-V25	*Vitis vinifera*	fruit	Verona/Vicenza

V-V26	*Vitis vinifera*	fruit	Verona/Vicenza
V-V27	*Vitis vinifera*	fruit	Verona/Vicenza
V-V28	*Vitis vinifera*	fruit	Verona/Vicenza
V-T1	*Vitis vinifera*	leaf	Trento
V-T2	*Vitis vinifera*	leaf	Trento
V-T3	*Vitis vinifera*	leaf	Trento
V-T4	*Vitis vinifera*	leaf	Trento
V-T5	*Vitis vinifera*	leaf	Trento
V-T6	*Vitis* sp.	leaf	Trento
V-T7	*Vitis* sp.	leaf	Trento
V-T8	*Vitis* sp.	leaf	Trento
V-T9	*Vitis* sp.	leaf	Trento
V-T10	*Vitis* sp.	leaf	Trento
V-T11	*Vitis vinifera*	fruit	Trento
V-T12	*Vitis vinifera*	fruit	Trento
V-T13	*Vitis lambrusca*	fruit	Trento
V-U1	*Vitis* sp.	leaf	Udine
V-U2	*Vitis* sp.	leaf	Udine
V-U3	*Vitis* sp.	leaf	Udine
V-U4	*Vitis* sp.	leaf	Udine
O-V29	*Olea europea*	fruit	Verona/Vicenza
O-V30	*Olea europea*	fruit	Verona/Vicenza
O-V31	*Olea europea*	fruit	Verona/Vicenza
O-V32	*Olea europea*	fruit	Verona/Vicenza
O-V33	*Olea europea*	fruit	Verona/Vicenza
O-V34	*Olea europea*	fruit	Verona/Vicenza
O-V35	*Olea europea*	fruit	Verona/Vicenza
O-V36	*Prunus armeniaca*	leaf	Verona/Vicenza
O-V37	*Pruns armeniaca*	leaf	Verona/Vicenza
O-V38	*Prunus armeniaca*	fruit	Verona/Vicenza
O-V39	*Prunus avium*	leaf	Verona/Vicenza
O-V40	*Malus domestica*	leaf	Verona/Vicenza
O-V41	*Malus domestica*	fruit	Verona/Vicenza
O-V42	*Cydonia oblonga*	fruit	Verona/Vicenza
S-V43	*Phytolacca americana*	infructescence	Verona/Vicenza

S-V44	*Phytolacca americana*	infructescence	Verona/Vicenza
S-V45	*Ruscus aculeatus*	fruit	Verona/Vicenza
S-V46	*Ruscus aculeatus*	fruit	Verona/Vicenza
S-V47	*Ruscus aculeatus*	fruit	Verona/Vicenza
S-V48	*Ruscus aculeatus*	fruit	Verona/Vicenza
S-V49	*Crataegus monogyna*	fruit	Verona/Vicenza
S-V50	*Crataegus monogyna*	fruit	Verona/Vicenza
S-V51	*Crataegus monogyna*	fruit	Verona/Vicenza
S-V42	*Rubus fruticosus*	fruit	Verona/Vicenza
S-V53	*Rosa canina*	fruit	Verona/Vicenza
S-Ta1	*Smilax axpera*	leaf	Taranto
S-Ta2	*Smilax aspera*	leaf	Taranto
S-Ta3	*Smilax axpera*	leaf	Taranto
S-Ta4	*Smilax aspera*	leaf	Taranto
S-Ta5	*Smilax aspera*	leaf	Taranto
S-Ta6	*Salvia rosmarinus*	leaf	Taranto
S-Ta7	*Salvia rosmarinus*	leaf	Taranto
S-Ta8	*Gineprus oxycedrus*	leaf and fruit	Taranto
S-Ta9	*Gineprus oxycedrus*	leaf and fruit	Taranto
S-Ta10	*Carpobrotus edulis*	leaf	Taranto
S-T14	*Capsicum annuum*	fruit	Trento
S-T15	*Capsicum annuum*	fruit	Trento
S-T16	*Solanum lycopersicum*	fruit	Trento

[1]code of isolate: first letter indicates the source (V vineyard, O orchard and S spontaneous or horticultural plant); second letter, after dash, indicates the location (V Verona-Vicenza, T Trento, U Udine and Ta Taranto) followed by progressive numeration for each location.

Table S2. Properties and classification of fungicides used in this study**.**

Trade name	Company	Formulation	Active ingredient (%)	Group name	Chemical group	MOA code	FRAC code	Propriety	Target disease
Folpan® 80 WDG	Adama Italia	water-soluble granules	folpet (80.0)	multi-site contact activity	phthalimide	M	M04	foliar	downy mildew
Carson® 45 WG	Adama Italia	water-soluble granules	cymoxanil (45.0)	unknown	cyanoacetamide-oxime	U	27	translaminar	downy mildew
Lidal®	Corteva agriscience	emulsifiable concentrate	tetraconazole (3.85)	demethylation inhibitor	triazole	G1	3	systemic	powdery mildew
Tucana® 25 EC	BASF Italia	emulsifiable concentrate	pyraclostrobin (23.6)	quinone oustide inhibitor	methoxy-carbamate	C3	11	foliar, translaminar	powdery mildew
Cantus®	BASF Italia	water-soluble granules	boscalid (50.0)	succinate-dehydrogenase inhibitor	pyridine-carboxamide	C2	7	foliar, translaminar	secondary rots
Prolectus® 50 WG	Sumitomo chemical Italia	water-soluble granules	fenpyrazamine (50.0)	keto reductase inhibitor	aminopyrazolinone	G3	17	translaminar	secondary rots

Figure S1. UPGMA dendrogram obtained by analysis of PCR-fingerprinting using primer $(GTG)_5$ and gel profiles of isolates belonging to genera *Rhodotorula* (A), *Sporidiobolus*/*Sporobolomyces* (B), *Curvibasidium* (C), *Filobasidium* (D), *Vishniacozyma* (E) and *Papiliotrema* (F).

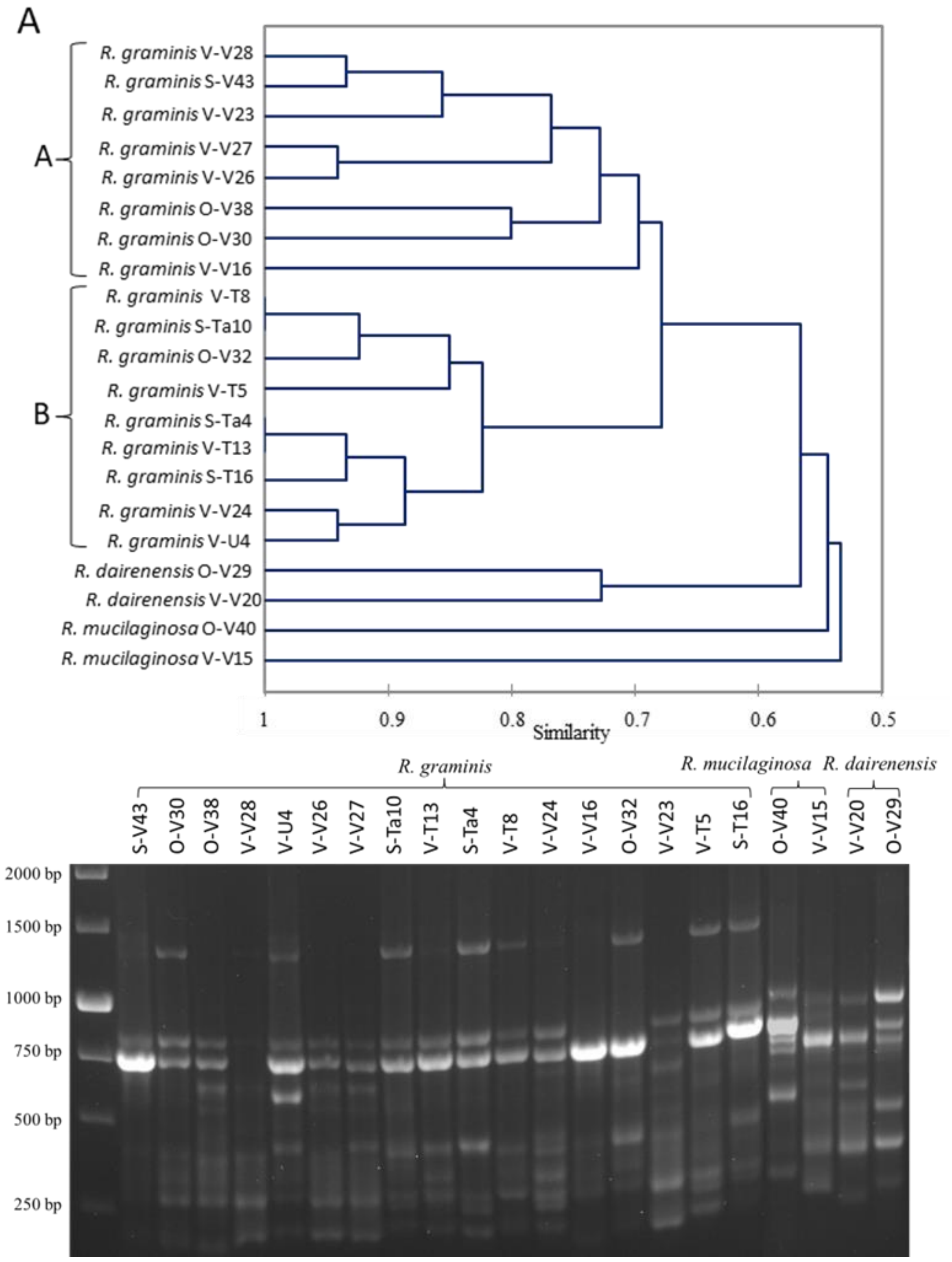

B

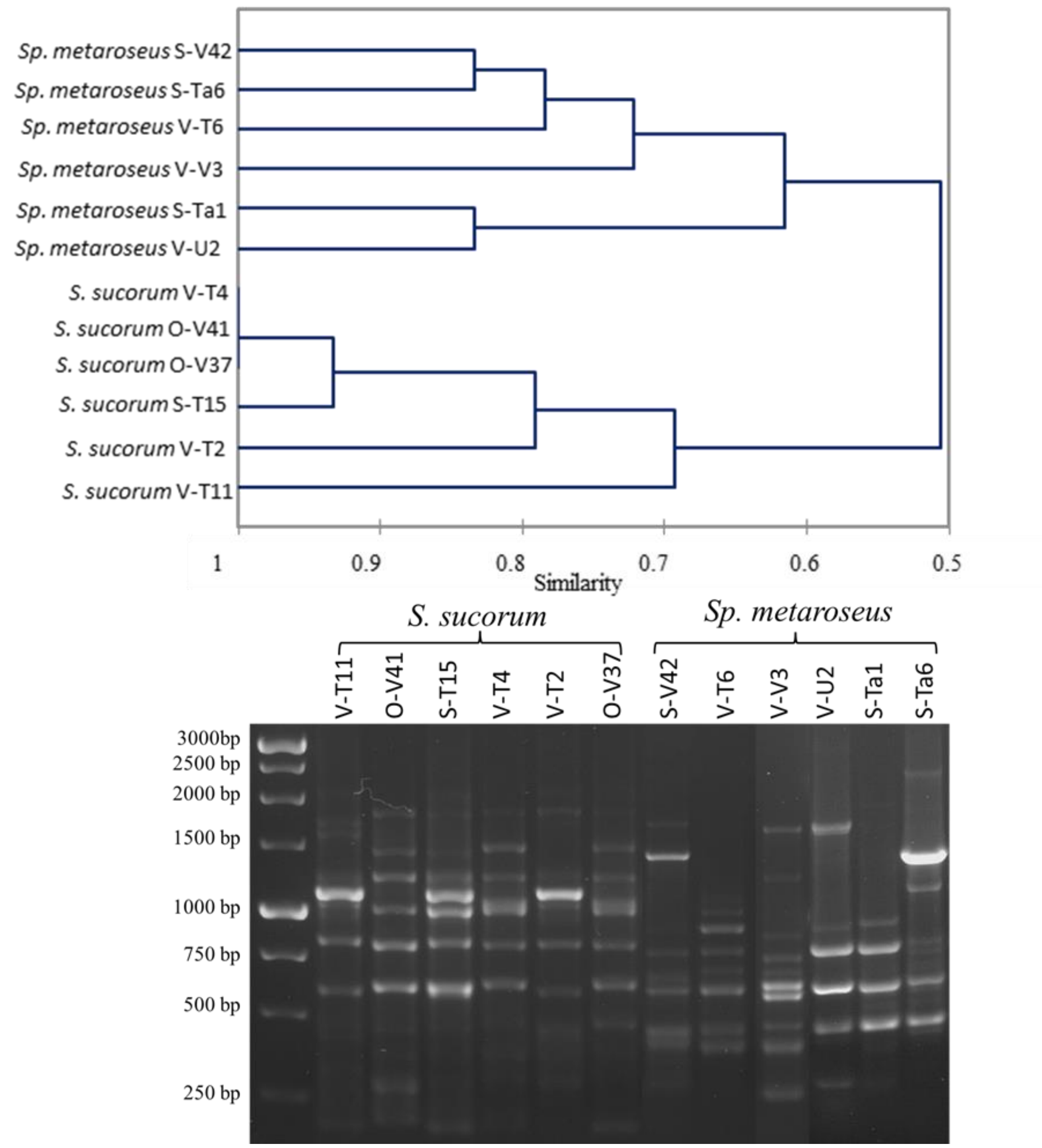

Sp. metaroseus S-V42
Sp. metaroseus S-Ta6
Sp. metaroseus V-T6
Sp. metaroseus V-V3
Sp. metaroseus S-Ta1
Sp. metaroseus V-U2
S. sucorum V-T4
S. sucorum O-V41
S. sucorum O-V37
S. sucorum S-T15
S. sucorum V-T2
S. sucorum V-T11
1
0.9
0.8
0.7
0.6
0.5
Similarity
S. sucorum
Sp. metaroseus
V-T11
O-V41
S-T15
V-T4
V-T2
O-V37
S-V42
V-T6
V-V3
V-U2
S-Ta1
S-Ta6
3000bp
2500 bp
2000 bp
1500 bp
1000 bp
750 bp
500 bp
250 bp

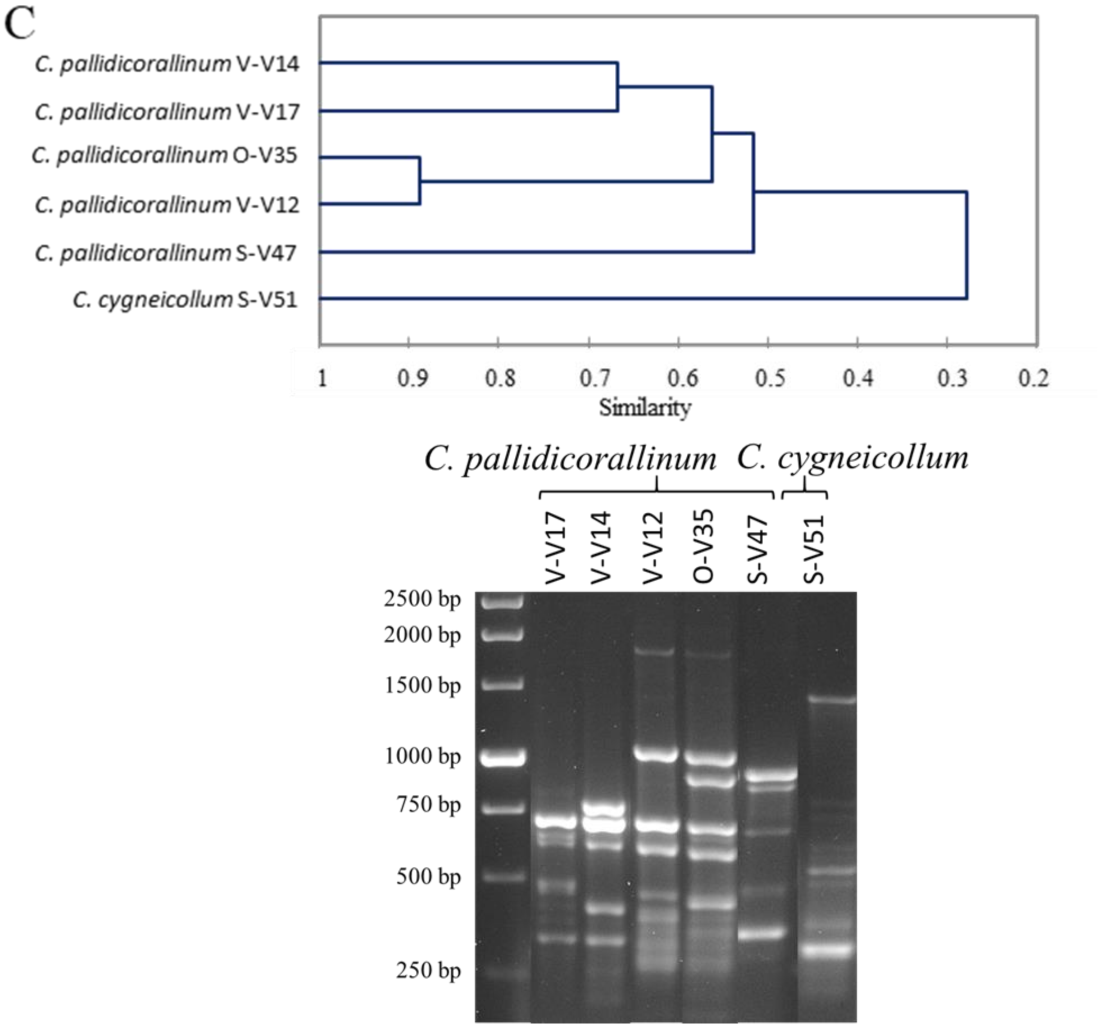
C
C. pallidicorallinum V-V14
C. pallidicorallinum V-V17
C. pallidicorallinum O-V35
C. pallidicorallinum V-V12
C. pallidicorallinum S-V47
C. cygneicollum S-V51
1
0.9
0.8
0.7
0.6
0.5
0.4
0.3
0.2
Similarity
C. pallidicorallinum
C. cygneicollum
V-V17
V-V14
V-V12
O-V35
S-V47
S-V51
2500 bp
2000 bp
1500 bp
1000 bp
750 bp
500 bp
250 bp

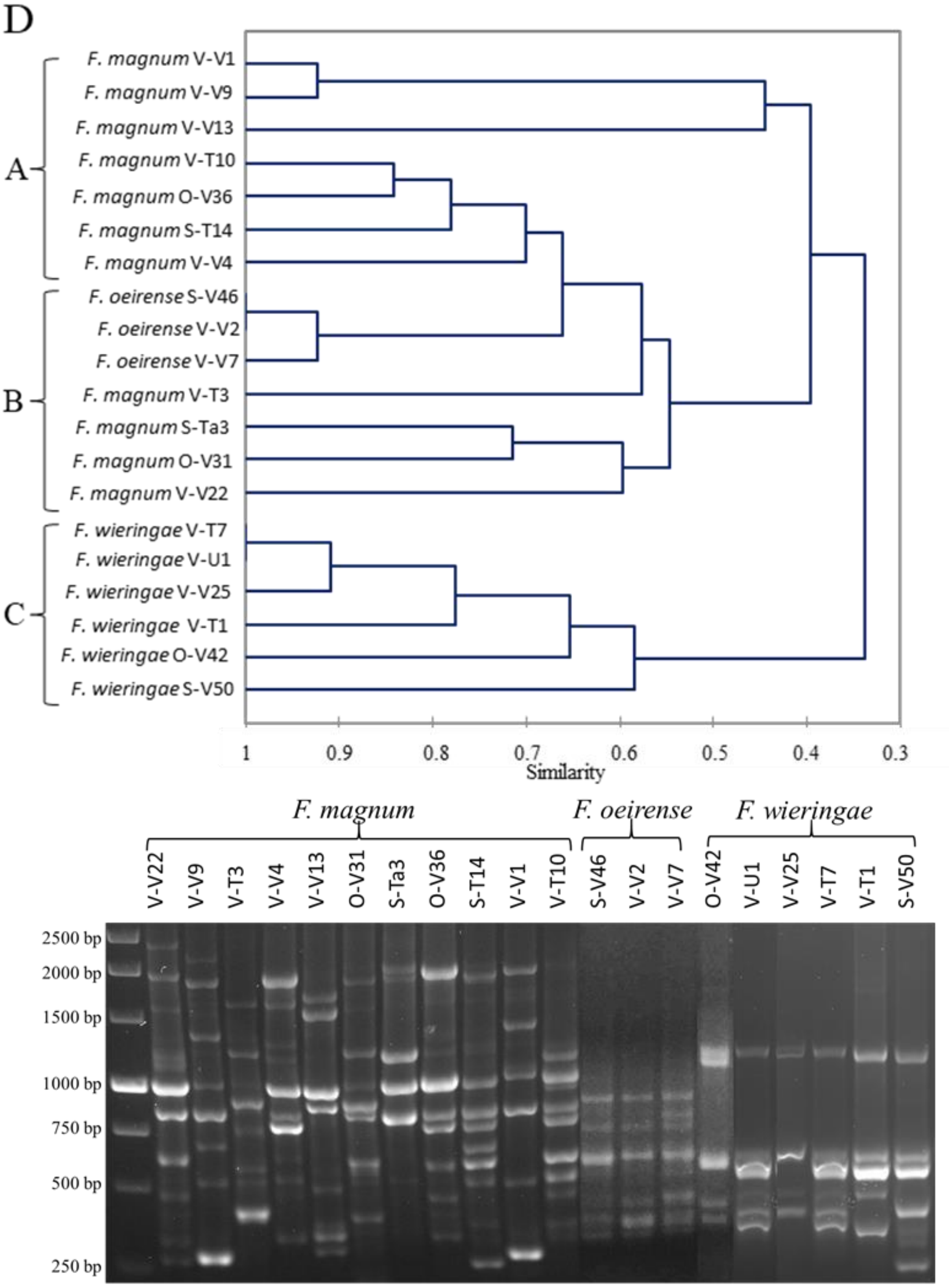

D
A
B
C
F. magnum V-V1
F. magnum V-V9
F. magnum V-V13
F. magnum V-T10
F. magnum O-V36
F. magnum S-T14
F. magnum V-V4
F. oeirense S-V46
F. oeirense V-V2
F. oeirense V-V7
F. magnum V-T3
F. magnum S-Ta3
F. magnum O-V31
F. magnum V-V22
F. wieringae V-T7
F. wieringae V-U1
F. wieringae V-V25
F. wieringae V-T1
F. wieringae O-V42
F. wieringae S-V50
1 0.9 0.8 0.7 0.6 0.5 0.4 0.3
Similarity
F. magnum
F. oeirense
F. wieringae
V-V22 V-V9 V-T3 V-V4 V-V13 O-V31 S-Ta3 O-V36 S-T14 V-V1 V-T10 S-V46 V-V2 V-V7 O-V42 V-U1 V-V25 V-T7 V-T1 S-V50
2500 bp
2000 bp
1500 bp
1000 bp
750 bp
500 bp
250 bp

E

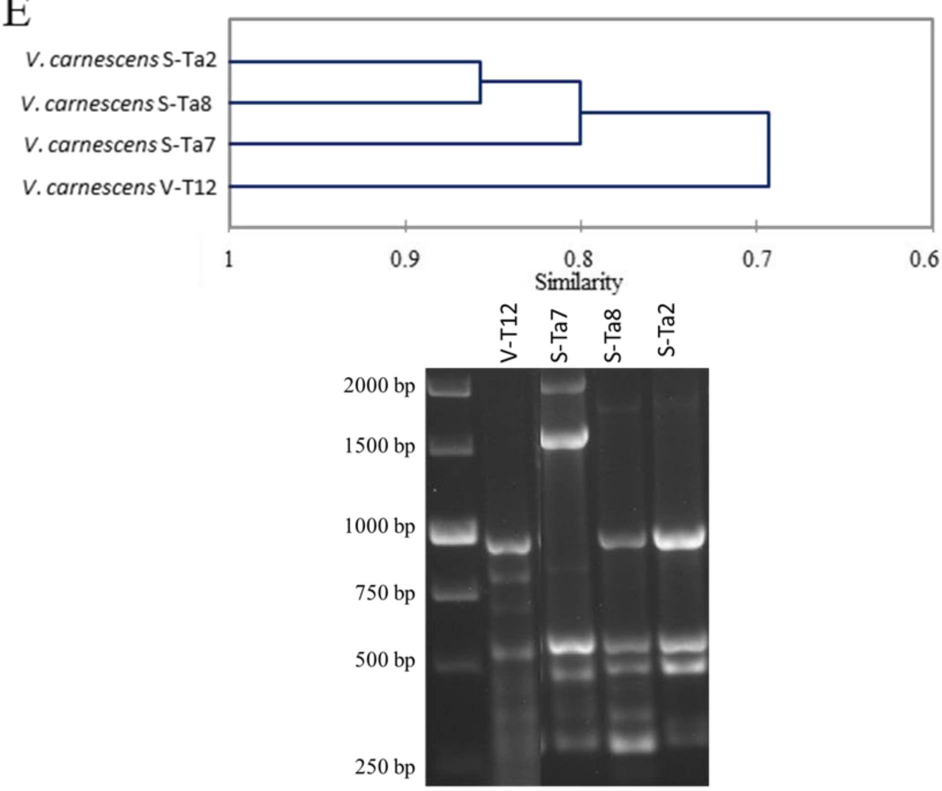
V. carnescens S-Ta2
V. carnescens S-Ta8
V. carnescens S-Ta7
V. carnescens V-T12
1
0.9
0.8
0.7
0.6
Similarity
V-T12
S-Ta7
S-Ta8
S-Ta2
2000 bp
1500 bp
1000 bp
750 bp
500 bp
250 bp

F

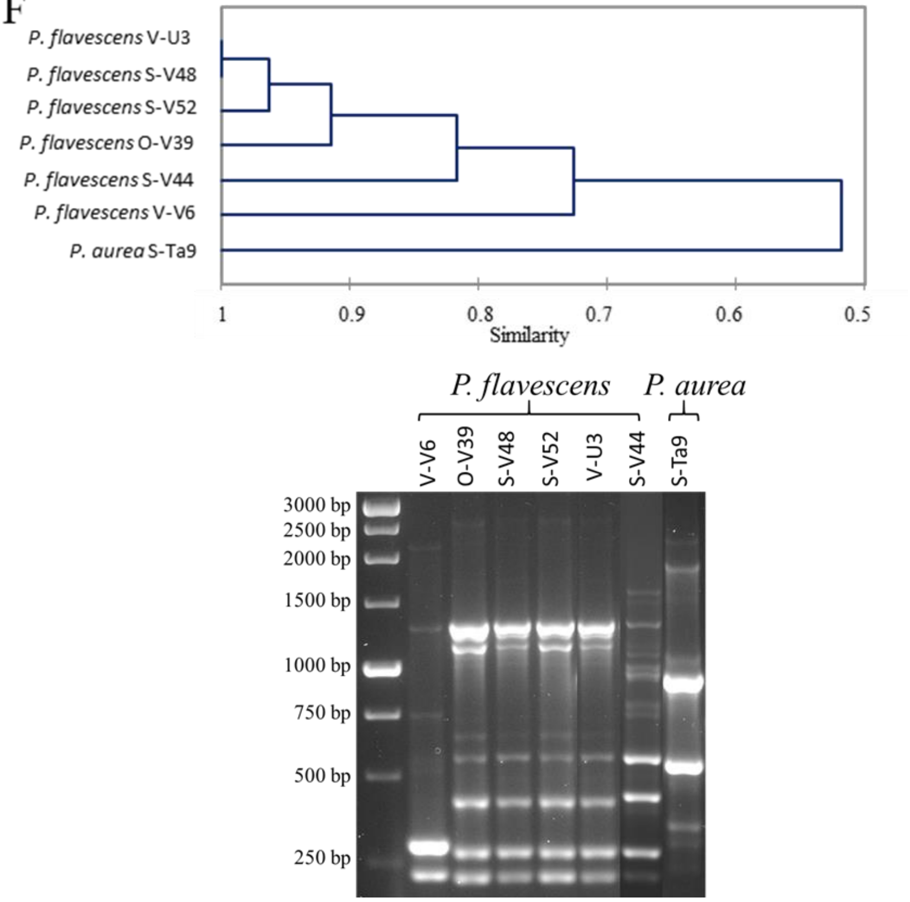
P. flavescens V-U3
P. flavescens S-V48
P. flavescens S-V52
P. flavescens O-V39
P. flavescens S-V44
P. flavescens V-V6
P. aurea S-Ta9
1
0.9
0.8
0.7
0.6
0.5
Similarity
P. flavescens
P. aurea
V-V6
O-V39
S-V48
S-V52
V-U3
S-V44
S-Ta9
3000 bp
2500 bp
2000 bp
1500 bp
1000 bp
750 bp
500 bp
250 bp

Table S3. Activity of extracellular hydrolytic enzymes (lipase L, protease P, *β*-glucosidase G, pectinase Pe, cellulase C and amylase A) of 83 yeast isolates determined by plate assay as high (h), moderate (m), low (l) and absent (a).

species	isolate	L	P	G	Pe	C	A
R. graminis	V-V16	h	h	m	a	a	a
R. graminis	V-V23	h	m	a	a	a	a
R. graminis	V-V24	h	l	m	a	a	a
R. graminis	V-V26	h	m	l	h	a	a
R. graminis	V-V27	h	m	l	a	a	a
R. graminis	V-V28	h	l	m	a	a	a
R. graminis	V-T5	h	h	m	a	a	a
R. graminis	V-T8	h	h	m	a	a	a
R. graminis	V-U4	h	m	l	a	a	a
R. graminis	V-T13	h	m	m	a	a	a
R. graminis	O-V30	h	m	l	a	a	a
R. graminis	O-V32	h	h	m	a	a	a
R. graminis	O-V38	h	h	m	a	a	l
R. graminis	S-V43	h	h	m	a	a	a
R. graminis	S-T16	h	m	l	a	a	a
R. graminis	S-Ta4	h	h	m	a	a	a
R. graminis	S-TA10	h	l	a	a	a	l
R. mucilaginosa	V-V15	m	a	a	a	a	a
R. mucilaginosa	O-V40	m	a	a	a	a	a
R. dairenensis	V-V20	l	a	a	a	a	a
R. dairenensis	O-V29	l	a	a	a	a	a
S. sucorum	V-T2	m	l	a	h	a	a
S. sucorum	V-T4	m	l	l	h	a	a
S. sucorum	V-T11	m	l	m	h	a	a
S. sucorum	O-V37	h	a	l	h	a	a
S. sucorum	O-V41	h	a	h	h	a	a
S. sucorum	S-T15	h	l	h	h	a	a
Sp. metaroseus	V-V3	h	h	a	h	a	a
Sp. metaroseus	V-V42	h	a	h	h	a	a
Sp. metaroseus	V-T6	h	l	l	h	a	a
Sp. metaroseus	V-U2	h	l	a	h	a	a
Sp. metaroseus	S-Ta1	h	a	a	l	a	a

Sp. metaroseus	S-Ta6	h	h	a	h	a	a
Rh. fluvialis	V-V5	m	a	h	a	a	a
Rh. colostri	S-V45	a	a	l	a	a	a
C. pallidicorallinum	V-V12	a	a	m	h	a	h
C. pallidicorallinum	V-V14	a	a	m	h	a	m
C. pallidicorallinum	V-V17	a	a	l	h	a	m
C. pallidicorallinum	O-V35	a	a	m	h	a	l
C. pallidicorallinum	S-V47	a	a	l	h	a	m
C. cygneicollum	S-V51	a	a	l	m	a	l
B. salicina	S-V49	m	a	h	a	a	a
Cy. slooffiae	S-Ta5	h	a	l	a	a	a
Sy. coprosmae	V-V10	m	a	h	a	m	a
F. magnum	V-V1	h	h	h	l	a	a
F. magnum	V-V4	h	h	m	l	a	a
F. magnum	V-V9	h	h	m	a	a	a
F. magnum	V-V13	h	h	h	l	a	a
F. magnum	V-V22	h	m	l	m	a	a
F. magnum	V-T3	l	m	m	m	a	l
F. magnum	V-T10	h	h	m	l	a	a
F. magnum	O-V31	h	h	m	l	l	m
F. magnum	O-V36	h	h	m	l	a	m
F. magnum	S-T14	h	h	m	m	a	l
F. magnum	S-Ta3	h	l	m	l	m	a
F. oeirense	V-V2	h	a	m	l	h	a
F. oeirense	V-V7	h	a	m	l	h	a
F. oeirense	S-V46	m	a	m	l	h	a
F. wieringae	V-V25	h	h	h	m	a	m
F. wieringae	V-T1	h	m	m	l	a	a
F. wieringae	V-T7	h	m	m	l	a	a
F. wieringae	V-U1	h	h	h	l	a	a
F. wieringae	O-V42	h	h	h	l	h	a
F. wieringae	S-V50	h	h	h	m	a	l
P. flavescens	V-V6	h	h	a	l	a	a
P. flavescens	V-U3	h	h	l	h	a	l
P. flavescens	O-V39	h	h	a	l	a	a
P. flavescens	S-V44	h	m	a	l	a	a
P. flavescens	S-V48	h	h	a	l	a	a
P. flavescens	S-V52	h	h	a	l	a	a

P. aurea	S-Ta9	h	h	l	l	a	a
V. carnescens	V-T12	m	a	l	a	m	l
V. carnescens	S-Ta2	a	a	a	a	m	l
V. carnescens	S-Ta7	m	a	l	l	a	a
V. carnescens	S-Ta8	a	a	a	a	m	a
K. mangroviensis	O-V33	l	l	l	h	m	l
K. mangroviensis	O-V34	a	a	l	m	l	a
K. pini	V-T9	m	a	a	m	a	l
N. diffluens	V-V21	h	l	l	a	a	a
H. sinensis	V-V11	m	a	h	a	a	a
T. ahsaii	V-V19	m	a	h	a	a	a
Ps. prolifica	V-V18	h	h	h	l	l	m
M. megachiliensis	V-V8	a	a	m	a	a	a

Table S4. Sensitivity to six commercial formulations of synthetic fungicides (Folpan® F, Carson® C, Lidal® L, Tucana® T, Cantus® Ca, Prolectus® P) of 83 yeast isolates assayed on plate as resistant (r), weakly sensitive (ws), sensitive (s) and highly sensitive (hs).

		F	C	L	T	Ca	P
R. graminis	V-V16	hs	hs	hs	ws	r	r
R. graminis	V-V23	hs	hs	hs	r	r	r
R. graminis	V-V24	r	s	s	ws	r	r
R. graminis	V-V26	r	hs	hs	r	r	r
R. graminis	V-V27	r	hs	r	r	r	r
R. graminis	V-V28	r	r	r	r	r	r
R. graminis	V-T5	r	d	d	r	r	r
R. graminis	V-T8	hs	hs	hs	r	r	r
R. graminis	V-T13	r	s	hs	r	r	r
R. graminis	V-U4	hs	hs	hs	r	r	r
R. graminis	O-V30	r	hs	s	r	r	r
R. graminis	O-V32	hs	hs	hs	r	r	r
R. graminis	O-V38	hs	hs	r	r	r	r
R. graminis	S-V43	hs	hs	hs	r	r	r
R. graminis	S-T16	r	ws	ws	r	r	r
R. graminis	S-Ta4	hs	r	hs	r	r	r
R. graminis	S-Ta10	hs	hs	hs	r	r	r
R. mucilaginosa	V-V15	hs	hs	s	r	r	r
R. mucilaginosa	O-V40	hs	hs	s	r	r	r
R. dairenensis	V-V20	hs	hs	hs	r	r	r
R. dairenensis	O-V29	hs	hs	hs	r	r	r
S. sucorum	V-T2	r	hs	ws	r	r	r
S. sucorum	V-T4	r	hs	r	r	r	r
S. sucorum	V-T11	r	hs	r	r	r	r
S. sucorum	O-V37	r	hs	ws	ws	r	r
S. sucorum	O-V41	ws	hs	r	s	r	r
S. sucorum	S-T15	r	hs	r	r	r	r
S. metaroseus	V-V3	hs	hs	hs	hs	r	r
S. metaroseus	V-V42	hs	hs	hs	hs	r	r
S. metaroseus	V-T6	hs	hs	hs	hs	r	r
S. metaroseus	V-U2	hs	hs	s	r	r	r
S. metaroseus	S-Ta1	hs	hs	hs	hs	r	r

S. metaroseus	S-Ta6	hs	hs	hs	hs	r	r
Rh. fluvialis	V-V5	hs	hs	hs	hs	r	r
Rh. colostri	S-V45	hs	hs	hs	hs	r	r
C. pallidicorallinum	V-V12	hs	hs	hs	ws	r	r
C. pallidicorallinum	V-V14	hs	hs	hs	s	r	r
C. pallidicorallinum	V-V17	hs	hs	hs	hs	r	r
C. pallidicorallinum	O-V35	hs	hs	hs	hs	r	r
C. pallidicorallinum	S-V47	hs	hs	hs	hs	r	r
C. cygneicollum	S-V51	hs	hs	hs	hs	r	r
B. salicina	S-V49	hs	hs	hs	hs	hs	r
Cy. slooffiae	S-Ta5	hs	hs	hs	hs	r	r
Sy. coprosmae	V-V10	hs	hs	hs	hs	hs	ws
F. magnum	V-V1	hs	hs	hs	hs	r	r
F. magnum	V-V4	hs	hs	hs	hs	r	r
F. magnum	V-V9	hs	hs	hs	hs	r	r
F. magnum	V-V13	hs	hs	hs	hs	r	r
F. magnum	V-V22	hs	hs	hs	hs	r	r
F. magnum	V-T3	hs	hs	hs	hs	r	r
F. magnum	V-T10	hs	hs	hs	s	r	r
F. magnum	O-V31	r	s	hs	ws	r	r
F. magnum	O-V36	hs	s	hs	hs	r	r
F. magnum	S-Ta3	hs	hs	hs	hs	r	r
F. magnum	S-T14	s	hs	hs	hs	r	r
F. oeirense	V-V2	hs	hs	hs	hs	ws	r
F. oeirense	V-V7	hs	hs	hs	hs	s	r
F. oeirense	S-V46	hs	hs	hs	hs	ws	ws
F. wieringae	V-V25	hs	hs	hs	hs	r	r
F. wieringae	V-T1	hs	hs	hs	hs	r	r
F. wieringae	V-T7	hs	hs	hs	hs	r	r
F. wieringae	V-U1	hs	hs	hs	hs	ws	s
F. wieringae	O-V42	hs	hs	hs	hs	r	r
F. wieringae	S-V50	hs	hs	hs	hs	r	r
P. flavescens	V-V6	hs	s	hs	s	r	r
P. flavescens	V-U3	hs	ws	hs	hs	r	r
P. flavescens	O-V39	hs	ws	hs	hs	r	r
P. flavescens	S-V44	hs	ws	hs	hs	r	r
P. flavescens	S-V48	hs	ws	hs	s	r	r
P. flavescens	S-V52	hs	ws	hs	hs	r	r

P. aurea	S-Ta9	hs	ws	hs	hs	r	r
V. carnescens	V-T12	hs	hs	hs	s	r	r
V. carnescens	S-Ta2	hs	hs	hs	hs	r	r
V. carnescens	S-Ta7	hs	hs	hs	hs	r	r
V. carnescens	S-Ta8	hs	hs	hs	hs	r	r
K. mangroviensis	O-V33	hs	hs	hs	hs	r	r
K. mangroviensis	O-V34	hs	hs	hs	ws	r	r
K. pini	V-T9	hs	ws	hs	hs	r	r
N. diffluens	V-V21	hs	ws	hs	hs	r	r
H. sinensis	V-V11	hs	ws	hs	hs	s	r
T. ahsaii	V-V19	s	r	hs	s	r	r
Ps. prolifica	V-V18	hs	hs	hs	hs	r	r
M. megachiliensis	V-V8	hs	hs	hs	r	r	r
